# YAP Regulates S-Phase Entry in Endothelial Cells

**DOI:** 10.1371/journal.pone.0117522

**Published:** 2015-01-30

**Authors:** Zhewei Shen, Ben Z. Stanger

**Affiliations:** 1 Department of Medicine, Gastroenterology Division, Perelman School of Medicine, University of Pennsylvania, Philadelphia, Pennsylvania, United States of America; 2 Abramson Family Cancer Research Institute, Perelman School of Medicine, University of Pennsylvania, Philadelphia, Pennsylvania, United States of America; 3 Department of Cell and Developmental Biology, Perelman School of Medicine, University of Pennsylvania, Philadelphia, Pennsylvania, United States of America; University of Minnesota, UNITED STATES

## Abstract

The Hippo pathway regulates cell proliferation and apoptosis through the Yes-associated protein (YAP) transcriptional activator. YAP has a well-described role in promoting cell proliferation and survival, but the precise mechanisms and transcriptional targets that underlie these properties are still unclear and likely context-dependent. We found, using siRNA-mediated knockdown, that YAP is required for proliferation in endothelial cells but not HeLa cells. Specifically, YAP is required for S-phase entry and its absence causes cells to accumulate in G1. Microarray analysis suggests that YAP mediates this effect by regulating the transcription of genes involved in the assembly and/or firing of replication origins and homologous recombination of DNA. These findings thus provide insight into the molecular mechanisms by which YAP regulates cell cycle progression.

## Introduction

The evolutionarily conserved Hippo pathway regulates cell proliferation, apoptosis, migration and cell fate specification through the YAP transcriptional regulator [1–5]. YAP itself has no DNA binding activity but rather associates with transcription factors—most notably TEA-domain (TEAD) factors—to regulate gene transcription in response to an upstream signaling cascade that involves tumor suppressors neurofibromatosis type II (Nf2), mammalian Ste-20 like kinases (Mst1/2), and large tumor suppressor (Lats1/2) [1,2,6,7]. High levels of Yap, achieved through mis-expression of YAP itself or deletion of upstream negative regulators, results in increased cell proliferation and carcinogenesis in many tissues [6–15]. In the liver, induction of a constitutively active form of YAP results in reversible liver enlargement and eventual adenoma and carcinoma formation[14]. In addition, elevated levels of YAP have been detected in multiple human cancers, where it has been associated with poor prognosis in some cases [14,16–18].

Several studies have investigated the mechanisms of YAP-associated tissue overgrowth using genomic approaches. Microarray analysis of mouse Yap transgenic livers detected increased expression of multiple cell cycle regulators such as *c-Myc*, *cyclin D*, *cyclin B*, *cyclin E* and *CDK6*. Yap transgene induction also stimulated the expression of survival genes including *birc5* and *birc2* [14]. Genome-wide expression profiling conducted by Zhao et al. also identified a similar list of cell cycle regulator proteins and apoptosis inhibitors as potential YAP targets in MCF10A cells with stable expression of either wild type or constitutively active YAP 5SA mutants [4]. Furthermore, YAP chromatin immunoprecipitation (ChIP)-on-chip analysis indicated that YAP can bind directly to the promoters of a subset of the cell cycle regulators identified in the MCF10A microarray [4].Consistent with these two studies, Lu et al. found that *birc5* and *cyclin D1* were upregulated in murine livers with tissue-specific deletion of *mst1* and *mst2* [9]. In aggregate, these genomic studies suggest that there may be a direct connection between YAP and cell cycle regulation.

Interestingly, Mizuno et al. found that *YAP* knockdown in mesothelioma cell lines led to an accumulation of cells in G0 and G1 as well as a significant loss of S phase cells [19]. Furthermore, microarray studies of YAP-deficient mesothelioma cells identified candidate genes involved in cell cycle regulation. These array candidates are also transcriptional targets of Rb/E2F, indicating the possibility of YAP-E2F-Rb crosstalk [19]. Specifically, ChIP analysis conducted by Mizuno et al. demonstrated that YAP directly regulates the expressions of *CCND1* (*cyclin D1*) and *FOXM1* (*foxhead box m1*), which in turn regulate G1 progression and G1/S transition [19]. Significantly, Kapoor et al. recently reported binding of YAP and TEAD2 to the promoters of several E2F1 targets involved in cell cycle regulation in pancreatic ductal adenocarcinoma cells [20]. These studies are consistent with the hypothesis that YAP exerts a pro-proliferative activity by transcriptionally regulating components of the cell cycle machinery, akin to the well-studied cell cycle regulator E2F [19,20].

Here, we employed human umbilical vein endothelial cells (HUVECs) to characterize and clarify the role of YAP in proliferation control and cell cycle regulation. Using siRNA knockdown and cell cycle analysis tools, we discovered that a loss of YAP blocked cell proliferation without causing apoptosis. The endothelial cells displayed G1 accumulation and S loss after *YAP* knockdown, and synchronization studies demonstrated that YAP is required for S-phase entry. Furthermore, microarray analysis identified candidates with critical roles in mediating origin licensing, firing and DNA damage repair. Taken together, these results suggest that YAP controls HUVEC proliferation by regulating the transcription of genes important for the G1-to-S transition.

## Results

### YAP is required for HUVEC proliferation and cell cycle progression

To understand the physiological role of YAP in cell proliferation control, we chose endothelial cells as our *in vitro* system based upon the finding that defective yolk sac vasculogenesis is among the first defects observed in YAP null mice [21]. We thus examined YAP protein expression in a number of mouse and human cell lines and determined that YAP was expressed in all except mouse lung endothelial cells and was unresponsive to the presence of vascular endothelial growth factor (VEGF) (S1A Fig.). We selected human umbilical vein endothelial cells (HUVECs) for further study because of their availability and ease of use.

**Fig 1:**
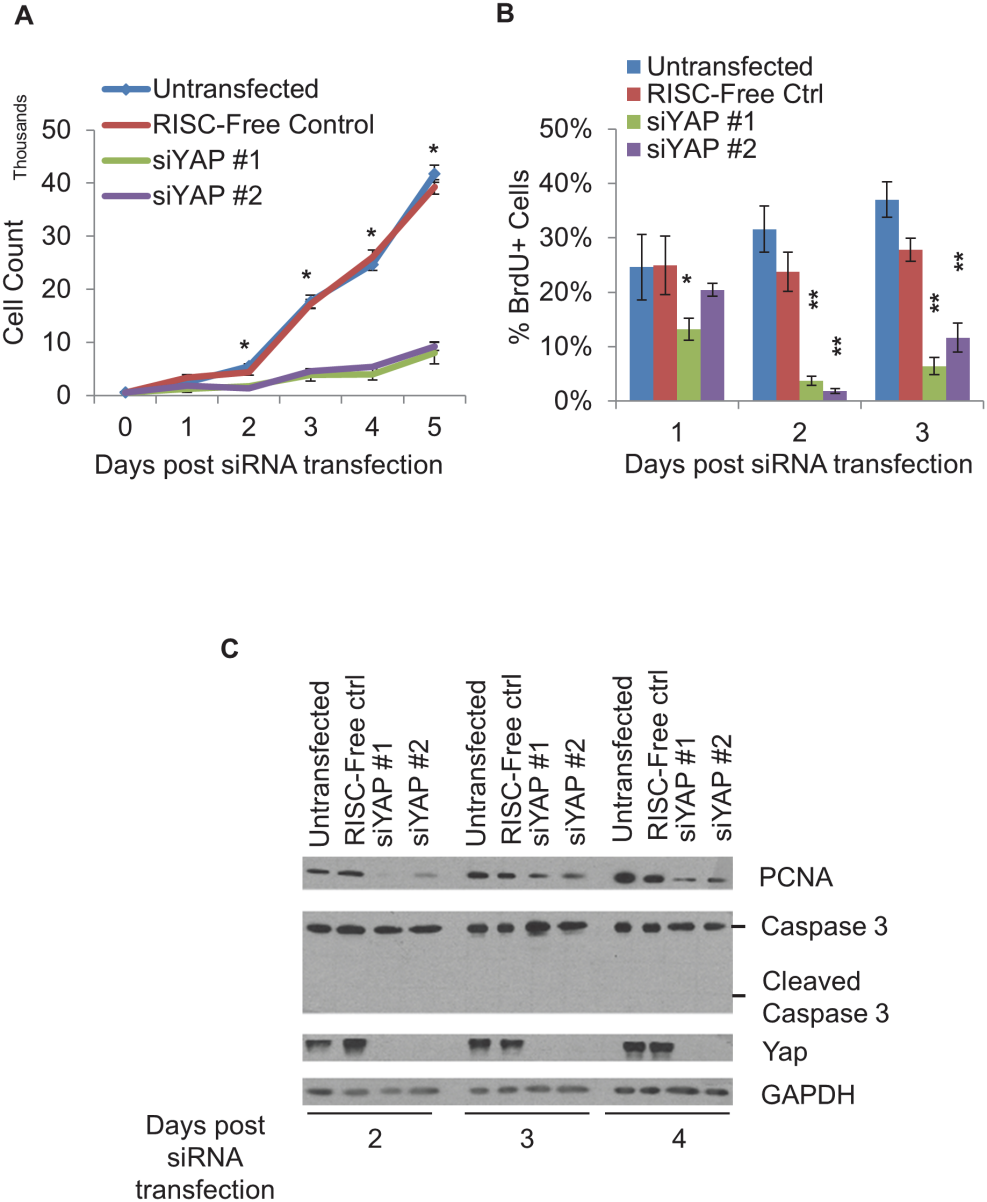
Deletion of *YAP* results in decreased HUVEC cell number. A. HUVEC cell number plotted against time after siRNA transfection. Untransfected and RISC-free siRNA were the control groups. Cells were counted on an Accuri C6 flow cytometer at 24 h intervals after transfection. There was a significant decrease in cell number in siYAP treated HUVECs compared to control HUVECs 48 h post-knockdown and at later time-points. Data are plotted as mean +/- standard error. N = 3 for each timepoint; *, p < 0.001. B. Quantification of BrdU+ HUVECs upon siYAP transfection. Cells were incubated with BrdU for 30 min and then stained with a BrdU antibody for visualization and quantification (plotted as the ratio of BrdU+ cells as a percent of total cells counted). Significantly fewer BrdU+ HUVECs were detected in siYAP-treated cells. Error bars indicate standard errors. P-values reflect differences compared to RISC-free control. *, p < 0.04; **, p < 0.007. C. Immunoblot (IB) assessing the levels of PCNA, CASPASE3, cleavage CASPASE3 in control and *YAP* knockdown (YAP-KD) cells. Protein samples were collected on d 1, 2 or 3 post-siRNA transfection and analyzed by western blot. No CASPASE3 cleavage was detected on d 2, 3 or 4, suggesting that *YAP* knockdown did not affect cell survival. See also S1 and S2 Figs.

We then tested the ability of two independent siRNAs to knock down *YAP*. In both HUVECs and HeLa cells, siYAP#1 or siYAP#2 resulted in the efficient knock down of YAP protein within 24 hours, an effect that lasted for at least 72 hours (S1B–C Fig.). We then quantified cell number over 5 days in HUVEC cells transfected with siYAP#1, siYAP#2, or a RISC-free control siRNA, and then compared them to untransfected cells. As shown in Fig. 1A, cells treated with siYAP#1 or siYAP#2 exhibited a significant decrease in cell number over the 5d time-course. Interestingly, the forward scatter measurement (FSC) of siYAP treated HUVECs was significantly higher than that of the controls starting 48 hours post siRNA transfection, suggesting that YAP-KD cells were considerably larger (S2A Fig.). Significantly, the changes in FSC profiles coincided with the slowed proliferation in YAP-KD cells at Day 2 post siRNA transfection (Fig. 1A).

To distinguish whether the blunted cell number was caused by increased apoptosis or decreased cell division, we first investigated whether loss of *YAP* affected cell division in HUVECs. We found that the percentage of BrdU+ HUVECs, reflecting the population of cells in S-phase, was significantly decreased in YAP-KD cells (2–3%) compared to control cells (23–32%) at the 48 h and 72 h time-points (Figs. 1B and S2B). In addition, PCNA levels were also decreased in siYAP treated cells, which is consistent with the results of the BrdU experiments (Fig. 1C). Furthermore, we also examined apoptosis in the *Yap* knockdown (YAP-KD) group by assessing the level of CASPASE3 cleavage (Fig. 1C). We found no cleaved CASPASE3 protein between d 2 and d 4, suggesting that increased cell death was unlikely to be contributing to the decrease in cell number with YAP-KD. These results suggest that YAP is essential for cell cycle progression, but not survival, in HUVECs.

**Fig 2:**
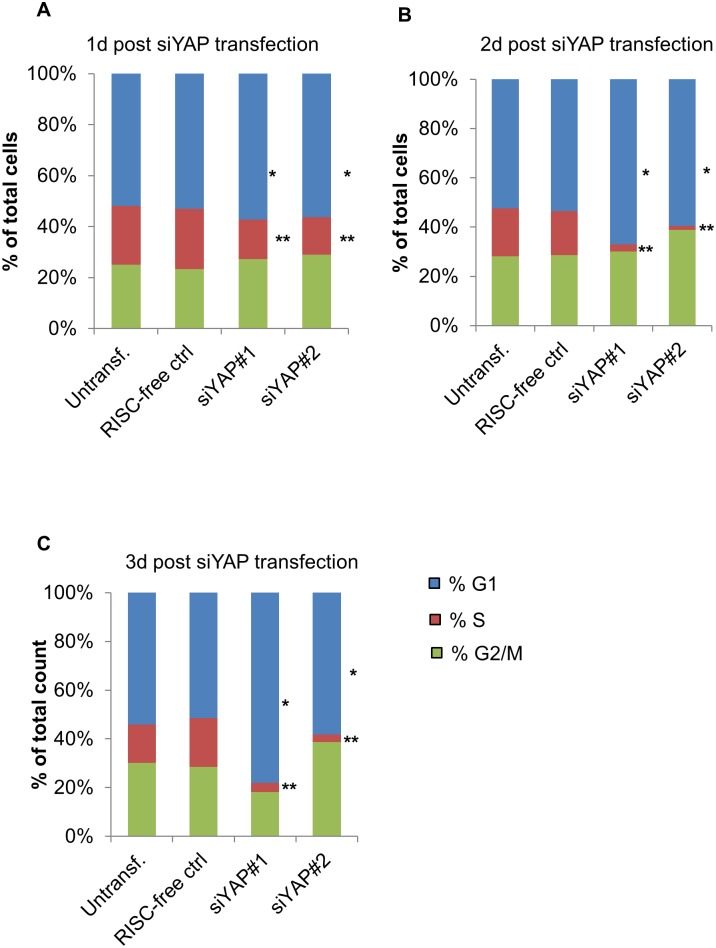
Yap knockdown in HUVECs results in G1 accumulation. A. Quantitative comparisons of cell cycle profiles comparing control and YAP-KD cells 1 d after siRNA transfection. Cells were incubated with BrdU for 1 hour and then stained for BrdU followed by FACS analysis. Data are plotted as the percentage of cells in each stage of the cell cycle (see S3 Fig.). HUVECs treated with siYAP exhibited a significant decrease in S-phase and increase in G1-phase at this time point. p-values were calculated by comparing each phase to the corresponding datasets from RISC-free control. *, p < 0.04; **, p < 0.007. B. Quantitative comparisons of cell cycle profiles between control and YAP-KD cells 2 d after siRNA transfection. Cells were processed and the data examined statistically as described in (A). *, p < 0.04; **, p < 0.007. C. Quantitative comparisons of cell cycle profiles between control and YAP-KD cells 3 d after siRNA transfection. Cells were processed and the data examined statistically as described in (A). *, p < 0.04; **, p < 0.007. See also S3 and S4 Figs.

We then used BrdU flow cytometry to establish detailed cell cycle profiles of siRNA-treated HUVECs at 24 h intervals (Figs. 2A-C and S3). Starting on day 1, YAP-KD cells began to exhibit marked S phase loss (Figs. 2A and S3). The phenotype was more pronounced at 2 d post-transfection, with control cells containing approximately six times more S-phase cells than the YAP-KD cells (18–23% vs. 2–3%) (Figs. 2B and S3), and persisted 3 d post-transfection (Figs. 2C and S3). Loss of S-phase was consistently associated with an increase in the percentage of cells in G1 but no change in the percentage of cells in G2/M (Fig. 2A-C). Thus, we conclude that the proliferation defects in HUVECs following *YAP* knockdown are the result of significant blockages in the G1 and/or S phases of the cell cycle.

We next sought to verify these effects on cell cycle in another cell line. For this purpose, we chose HeLa cells, in which we had previously shown efficient knockdown with siRNA (S1C Fig.). Surprisingly, treatment with siYAP#1 or siYAP#2 had no effect on HeLa cell number or cell cycle profile (S4A–B Fig. and data not shown). This result suggests that YAP regulates the cell cycle in a cell type-specific manner. We therefore performed all subsequent studies in HUVECs.

### YAP regulates S phase entry in HUVECs

**Fig 3:**
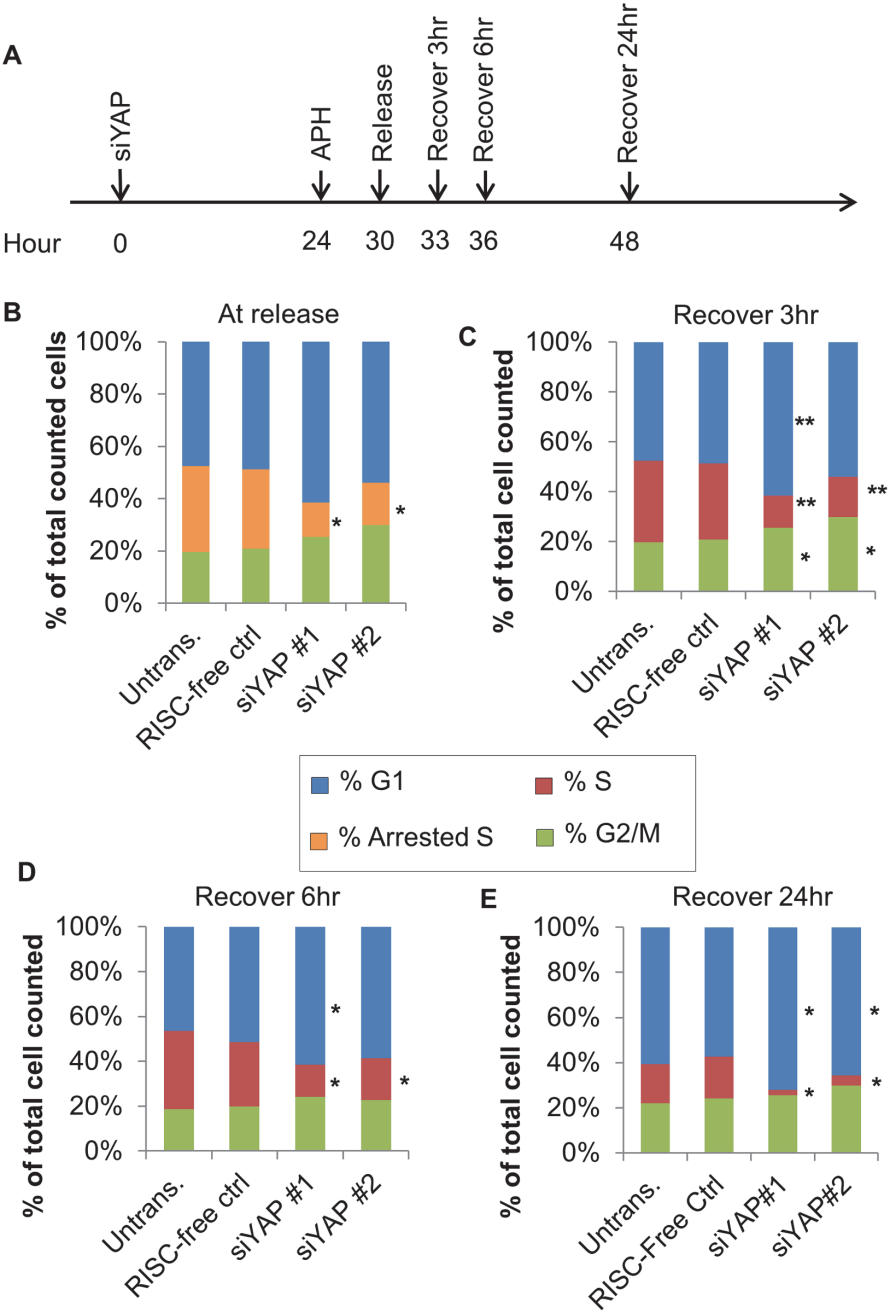
Yap is not required for S-phase progression in HUVECs. A. Schematic of the APH experiment. HUVECs were treated with siRNA for 24 hours then incubated with 5µM APH for 6 h. HUVECs were collected at 3, 6, or 24 h post-APH removal, receiving a pulse of BrdU at the time of harvest. B. Quantification of cell cycle profiles comparing control and YAP-KD cells after 6 h APH arrest (“Release”). All HUVECs exhibited S-phase stall upon APH treatment (orange bars). Significantly fewer S-phase cells were detected in siYAP-treated HUVECs. p-values were calculated by comparing each phase to the corresponding datasets from RISC-free control. *, p < 0.04. C. Quantification of cell cycle profiles comparing control and YAP-KD cells 3 hours after APH removal. All HUVECs, including siYAP-transfected cells, reinitiated S-phase with similar kinetics. p-values were calculated as described in (B). *, p < 0.04; **, p < 0.007. D. Quantification of cell cycle profiles comparing control and YAP-KD cells 6 h after APH removal. HUVECs with siYAP transfection could restart stalled S-phase in a YAP-independent manner. p-values were calculated as described in (B). *, p < 0.04. E. Quantification of cell cycle profiles comparing control and YAP-KD cells 24 h after APH removal. Normal cell cycle profiles were re-established in control cells while the YAP-KD cells had fewer S-phase cells, suggesting that S-phase progression is YAP independent. p-values were calculated as described in (B). *, p < 0.007. See also S5 Fig.

Because the G1 accumulation of YAP-KD cells may have resulted from blocks in G1 progression, S-phase entry or S-phase progression, we utilized aphidicolin (APH)—which inhibits DNA polymerase, thereby arresting S-phase—to distinguish between these scenarios [22,23]. We began by incubating HUVECs with APH for 6 hours and then characterized cell cycle recovery at 3, 6, and 24 hours after “release” in the presence or absence of the previously described siRNA (Fig. 3A). As expected, 6 h APH treatment temporarily blocked DNA replication, resulting in a subset of HUVECs stalled in S-phase; we refer to cells in this state as “arrested S” due to their high DNA content (between 2n and 4n) and low BrdU intensity (Figs. 3A-B and S5, upper left panel).

Upon APH removal, HUVECs in the “arrested S” group were expected to resume normal S phase, with the expectation that if YAP regulates S-phase progression, then YAP-KD HUVECs should exhibit defects in this recovery process. We thus compared the percent of cells in normal S phase (“S”) between controls and YAP-KD cells after 3, 6 and 24 hours of APH washout (Figs. 3C-E and S5 second graph in the first column). The “arrested S” subgroup was absent in both control and KD groups starting 3 hours post APH release, suggesting that YAP was not required for S-phase re-initiation (Figs. 3C and S5, red arrows). Furthermore, both control and YAP-KD cells continued to progress through S-phase at 6 hours of APH removal (Figs. 3D and S5, blue arrows). By 24 hours post APH washout, however, a significant loss of S-phase cells was observed in the YAP-KD groups (Fig. 3E). It is important to note that YAP-KD cells exhibited a significantly lower percentage of both arrested S (Fig. 3B) and normal S (Fig. 3C-E) which was likely the effect of YAP siRNA, not a result of APH treatment. These results demonstrate that YAP is not required for S-phase progression, as both control and YAP-KD HUVECs were equally capable of resuming the S phase after APH removal. Instead, our findings thus suggest that YAP is required at some point in the cell cycle prior to the initiation of S-phase.

**Fig 4:**
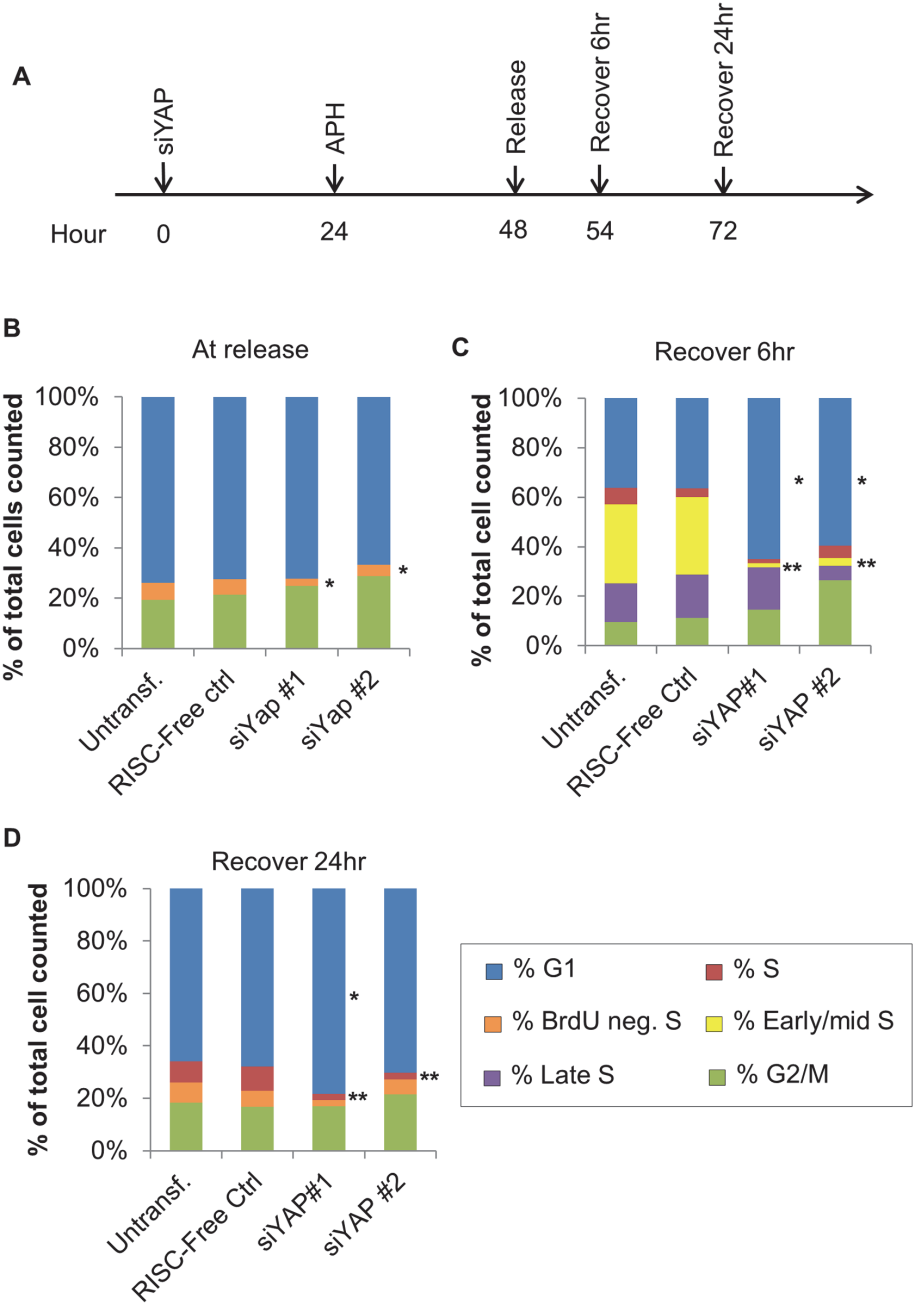
Yap is required for S-phase entry in HUVECs. A. Schematic of the APH experiment. HUVECs were treated with siRNA for 24 h then incubated with 5µM APH for 24 h. BrdU-treated HUVECs were collected at 3, 6, or 24 h post-APH removal. B. Quantification of cell cycle profiles comparing control and YAP-KD cells at the end of the 24 h APH arrest. Most of the cells captured by FACS were BrdU negative and had DNA contents of either 2N or 4N (reflecting arrest predominantly in G1 and G2/M). siYAP treatment under these conditions resulted in a further decrease of S-phase cells. p-values were calculated by comparing each phase to the corresponding datasets from RISC-free control. *, p < 0.04. C. Quantification of BrdU FACS cell cycle analysis 6 h after APH removal. Control cells were able to initiate S-phase indicated by “early S” cells. However, this population of cells was significantly reduced in siYAP-transfected cells. p-values were calculated as described in (B). * (blue bar, compared to the controls), p < 0.04. ** (yellow bar, compared to the controls), p< 0.007. D. Quantification of BrdU FACS cell cycle analysis 24 h after APH removal. siYAP-treated cells continued to exhibit decreased numbers of S-phase cells after 24 h of APH recovery. p-values were calculated as described in (B). * (blue bar, compared to the controls), p < 0.04; ** (red bar, compared to the controls), p < 0.007. See also S6 Fig.

To further define this cell cycle block, we incubated HUVECs with APH for a full 24 h with the expectation that this treatment would allow us to determine directly whether YAP-KD cells have a defect in S-phase entry (versus progression) (Fig. 4A). After prolonged exposure to APH, HUVECs were arrested predominantly in either G1 (2n DNA with low BrdU) or G2/M (4n DNA with low BrdU) (Figs. 4B and S6, upper left panel). A smaller group of cells containing low BrdU and a DNA content between 2n and 4n was also detected and termed “BrdU neg. S” to reflect a subset of HUVECs that were likely severely and permanently impacted by the extensive APH treatment (Figs. 4B and S5 upper left panel). Importantly, it is possible that the G1 and G2/M groups may also contain arrested S-phase cells with either 2n or 4n DNA, respectively.

In control cells, two populations of BrdU+ cells emerged within 6 h of APH release: cells with a DNA content between 2n and 4n, representing cells that had re-entered “early/mid S-phase,” and cells with 4n DNA, representing cells in “late S-phase” (Figs. 4C and S6). Under these conditions, YAP-KD cells had a near-normal “late S-phase,” likely reflecting arrested S-phase cells that had resumed DNA synthesis after APH washout (Figs. 4C and S6). By contrast, there was an almost complete loss of “early/mid S-phase” cells in YAP-KD cells (S6 Fig., red arrows). These results further indicate that YAP is required for S-phase entry but not S-phase progression in HUVECs.

### YAP regulates origin recognition and DNA repair pathways

To determine the YAP transcriptional targets that might mediate these effects, we conducted microarray analysis comparing YAP-KD and control cells. Because we wished to maximize the chances of identifying primary targets, we selected 30 h after transfection as our time-point for our analysis as there was a greater than 90% decrease in *YAP* mRNA with only a small decrease in cell proliferation at this time-point (Figs. 1–2 and S7A).

**Fig 5:**
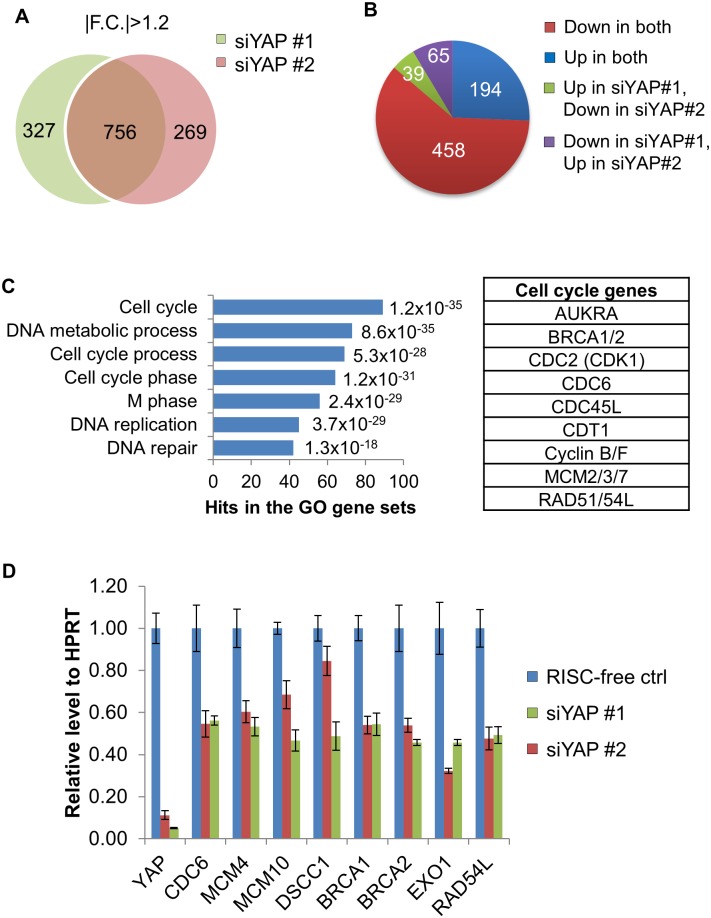
YAP regulates the expressions of factors critical for replication origin biology and homologous recombination. A. Venn diagram comparing microarray analysis results from HUVECs treated with either siYAP#1 or siYAP#2 for 30 h. Candidate genes with expression fold change of 1.2 or more, a p-value less than 0.05, and a false discovery rate (FDR) of less than 0.1 were used. 756 genes were common hits for the siRNA transfected cells, a number that corresponds to approximately 70% of the genes that exhibited expression changes with either siRNA. Fold changes were calculated using RISC-free control line as the reference group. Each condition contains four biological replicates (N = 4). B. Pie chart analysis of the 756 genes from (A). “Up” and “down” represent the directionality of expression changes of each gene in siYAP #1 or #2 treated HUVEC. Most of the 756 genes were down-regulated with both siRNAs (red group). Approximately 25% of genes were up-regulated with both siRNAs (blue group), while 14% showing discordant directionality of change (green and purple groups). C. DAVID bioinformatics gene ontology analysis of the top 8 hits/pathways within the red group from (B). The number of hits in each matching gene ontology group is graphed on the X-axis, with weighed p-values listed next to each histogram. Representative candidate genes with major roles in cell cycle regulation identified in the ontology analysis were listed in the “Cell cycle genes” table. D. RT-PCR validation of the microarray results. All candidates except DSCC1 exhibited significant expression changes in siYAP#1- and siYAP#2-treated cells. Transcript abundance was calculated by normalizing each measurement to an HPRT control. See also S7 and S8 Figs.

Using a cutoff of 1.2-fold expression change and a false-discovery rate of 0.1 or less, we found 756 genes that were differentially expressed in cells transfected with siYAP#1 and siYAP#2 as compared to the RISC-free control group (Fig. 5A, S1 Table). 458 of these 756 genes (approximately 61%) displayed decreased expression in the YAP-KD cells, consistent with the notion that YAP acts mainly as a transcriptional activator (Fig. 5B). A small fraction of the 756 genes (~14%) exhibited differential changes in expression between siYAP#1 and siYAP#2, which we attributed to the off-target effects of the siRNA system. Interestingly, approximately 25% of differentially-expressed genes had increased transcript abundance in the absence of YAP (Fig. 5B).

To narrow down the set of genes that would be subjected to further analysis we curated the gene list to include those exhibiting a greater than 1.5-fold change in transcript abundance with both siYAP#1 and siYAP#2 (S7B Fig., S2 Table). Using this higher fold-change cutoff, only 30–40% of the differentially expressed genes were “shared” by siYAP#1 or siYAP#2, raising the possibility of significant off-target effects. To address this possibility, we analyzed the microarray dataset in a step-wise fashion. We found that when the fold change threshold was set to 1.5 for siYAP #1 and 1.2 for siYAP#2, over 95% of the candidate genes were in the overlapping list for siYAP#1. A similar result was obtained when the reverse threshold was used (S7C Fig.). Thus, the 353 genes showing a greater than 1.5-fold expression change with either siRNA (of which 70 are differentially expressed in both) likely reflect the consequence of on-target YAP knockdown (with variable fold-change) rather than an off-target effect. Again, the majority of these 70 differentially-expressed genes were transcriptionally down-regulated with both siRNAs, while a small fraction of the genes were induced or discordantly regulated (S7D Fig.).

DAVID bioinformatics resource database and gene set enrichment analysis revealed that the loss of YAP negatively affected the expression of multiple genes critical to cell cycle, DNA replication and DNA damage repair processes (Fig. 5C) [24–27]. Specifically, we found that the expression of several genes involved in replication origin function (cell division cycle 6/CDC6, minichromosome maintenance protein/MCM4, MCM10) and homologous recombination (breast cancer 1/2/BRCA1/2, DNA repair and recombination protein 54-like/RAD54L, exonuclease 1/EXO1) were negatively affected in YAP-KD mutants [28,29]. Additional genes critical to origin licensing and firing (DNA replication factor 1/CDT1, cyclin dependent kinase 2/CDK2) as well as homologous recombination (RAD51) were identified as YAP targets when the fold change threshold was lowered to 1.2 fold (Fig. 5C) [28,29]. We confirmed that many of these targets were down-regulated at the transcript level by qPCR (Fig. 5D). Furthermore, western blotting with commercially available antibodies for two of the replication origin function factors (MCM4 and CDC6) also showed decreased expression of these proteins in YAP-depleted cells (S8 Fig.). Thus, the HUVEC proliferation defects observed following YAP knockdown are associated with decreased expression of genes involved in DNA replication origin function and DNA repair.

## Discussion

### YAP regulates HUVEC proliferation

Consistent with prior studies with mesothelioma cells [19], we found that YAP is required for cell cycle progression in human primary endothelial cells. BrdU analysis of YAP-KD HUVECs suggested that YAP is required during S-phase, and two sets of APH arrest experiments were conducted to distinguish the role of YAP in this process. The short-term APH arrest assessed the ability of YAP-KD mutants to reinitiate stalled S-phase. As both control and mutants groups recovered from the arrest with similar kinetics, we conclude that HUVECs can progress through S-phase independent of YAP. By contrast, YAP-KD cells were unable to begin S-phase following long-term APH block. Taken together, these APH synchronization experiments demonstrate that YAP controls cell proliferation at least partly by facilitating a G1-to-S transition.

Our results do not rule out the possibility that YAP could also act at other stages of the cell cycle. For example, although our microarray analysis did not identify G1 progression specific genes such as E2Fs, Rb or G1-phase cyclin and CDKs, YAP could be required for cells to move through the G1-phase of the cell cycle. Likewise, YAP could be acting during G2/M, a notion supported by the finding that the HUVEC microarray analysis identified several G2- and M-phase factors including *CDC25A*, *AURKA* (*AURORA KINASE A)* as genes whose expression was decreased with YAP-KD [30,31]. Nevertheless, as we did not detect reproducible alterations in G2/M progression in YAP-KD HUVECs, any effect on this phase of the cell cycle is likely to be small.

### Context-specific transcriptional regulation by the Hippo pathway

It is worth noting the variety and context-dependency of Hippo pathway target genes that have been reported [32–37]. Muramatsu et al. identified BIRC5 and CDKN2A/p21 as two significant targets of YAP that are responsible for modulating the survival and proliferation in KYSE170 cells [35]. In addition, the Hippo pathway target CTGF may also function as a driver of YAP-induced colon cell tumorigenesis [36]. Hao et al. reported that YAP can regulate multiple genes including BIRC5, ITGB2, IGFBP3 and p57 to promote survival and migration of MCF10A cells [37]. Interestingly, we did not detect any of these genes except BIRC5 in our HUVEC study, a discrepancy that may be the result of tissue-specific differences in YAP transcriptional outputs, since none of the aforementioned studies utilized HUVEC cells. Another reason that we identified a different set of YAP targets compared to other gene knockdown studies may involve differences in experimental design. Our study used an early time-point (30 h post-knockdown) in order to preferentially identify proximal targets, as opposed to other studies which examined cells between 48 h and 5 d after YAP knockdown [19,36]. Irrespective of these differences, the list of candidate genes we observed is most consistent with the one reported by Kapoor et al., which concluded that YAP cooperates with E2F1 in Kras induced pancreatic adenocarcinoma (PDAC) by modulating a large subset of E2F1 targets including Cdc6, Cdk1, Mcm complex components and Rad51 [20]. Taken together with the results of Kapoor et al., our study suggests that YAP regulates HUVEC cell proliferation via a specific set of E2F1-regulated cell cycle determinants.

### YAP controls S-phase entry by regulating the DNA replication machinery

Our microarray analysis suggested that the cell cycle defects following YAP-KD could be due to faulty licensing and/or firing of replication origins. Origin licensing involves the recruitment of a multi-protein complex (CDC6, CDT1 and MCM2–7) to DNA replication origins in a step-wise fashion [38–40]. YAP knockdown in HUVECs caused a reduction in, but not complete absence of, *CDC6*, *CDT1*, *MCM4* and *MCM10*, a finding that would be expected to result in a prolonged S-phase (secondary to a reduction in the number of origins). Instead, the YAP-KD phenotype is more consistent with defects related to origin firing, which marks the initiation of DNA synthesis and S-phase entry [38,41,42]. Even though origin firing is dependent on origin licensing, this process involves an overlapping set of factors including MCM2–7, CDC45, CDC7/DBF4 and S-phase CDK [28,41,43,44], without which S-phase cannot begin [28,41,43,44]. Significantly, our microarray identified MCM4, MCM10 and CDK2 as genes differentially expressed following YAP-KD. Although this result suggests that YAP is critical for origin firing, the fact that the microarray analysis identified both licensing and firing genes leaves open the possibility that YAP could play a significant role in both processes.

Interestingly, many of the differentially-expressed genes identified by microarray are also targets of E2F proteins [28,39,43,45,46]. Kapoor et al. recently demonstrated that YAP and E2F1 converge on a subset of target gene promoters in Kras-dependent pancreatic ductal adenocarcinoma [20]. Dominant negative E2F1 can mitigate the oncogenic effect of YAP in colony formation experiments [20]. In addition, ChIP-Seq experiments have identified YAP promoters as potential targets of several E2F proteins [47]. These data thus raise the possibility of crosstalk between the Hippo and E2F pathways in proliferation control.

One other notable observation is that approximately 25% of all differentially-expressed genes with an expression fold change of 1.2-fold or above following YAP-KD were up-regulated (Fig. 5B). Although these genes may not be direct YAP targets, reflecting instead a secondary response to YAP-KD, it is also possible that YAP may possess repressor functions that have yet to be described. Consistent with this notion, Zhao et al. also identified a substantial number of down-regulated genes in MCF10A cells with ectopic YAP [4], and in the future it will be important to determine whether YAP has both activating and repressive activities.

### DNA damage repair and YAP

Our microarray analysis also identified several factors involved in homologous recombination (HR) [29,48,49]. The HR pathway is a high-fidelity mechanism for fixing double stranded DNA breaks (DSB) using homologous templates. Among the differentially-expressed genes following YAP-KD were factors critical for the process of DNA strand invasion into the homologous chromosomes for repair, including BRCA1/2, RAD54L, EXO1 and RAD51 [29,48,49]. This result suggested that YAP may facilitate the repair of DSBs, a common event during S-phase, by regulating the core HR machinery. Notably, re-introduction of *YAP* in Jurkat and OCI/AML3 cells reinstated the cellular apoptotic programs upon DNA damage signals [50]. Loss of BRCA1/2 is known to lead to a higher predisposition to human breast and ovarian cancers [51–55]. Interestingly, studies of BRCA in non-small-cell lung cancer demonstrated that overabundance of BRCA is associated with more aggressive cancer [56]. Because YAP is elevated in many human tumors, including lung and ovarian cancers, YAP may play factor into the biology of these tumors as a regulator of HR genes in addition to its direct effects on the cell cycle.

### YAP and apoptosis

Numerous studies have demonstrated that Yap, in both *Drosophila* and mammals, exerts an anti-apoptotic effect [1,14,57]. For example, Yap transgenic livers are less sensitive to the effects of cell death inducers, possibly because Yap stimulates the expressions of survival genes including Survivin/Birc5 [5,14,19,20,58]. Despite the fact that our microarray analysis detected decreased expression of BIRC5 expression in YAP-KD cells (S1 Table), we did not detect any significant sign of apoptosis in siYAP#1 or siYAP#2 treated cells. Our findings thus suggest that in HUVECs, YAP is not required for survival. However, it will be interesting to determine whether loss of YAP will affect HUVEC cells following a death-inducing challenge.

## Materials and Methods

### Cell culture and cell growth curve

Human umbilical vein endothelial cells (HUVEC, CC-2517) were commercially purchased (Lonza, Allendale, NJ) and maintained on plates coated with 0.1% gelatin (Fisher Scientific, Pittsburgh, PA) with supplemented EGM2 media (Lonza). HeLa cells were maintained in 10% FBS (GE Healthcare, Logan, UT) and 1% Pen/Strep (Life Technology, Grand Island, NY). For aphidicolin arrest, HUVEC was treated with 5µM aphidicolin (EMD Millipore, Billerica, MA) for either 6 hour or 24 hour and then reintroduced to EGM2 media after PBS wash.

HUVECs were trypsinized and collected every 24 hours after siRNA transfection. Cell numbers were counted using Accuri C6 immediately after collection (BD Biosciences, San Jose, CA). Forward scatter and size scatter values were collected with cell count simultaneously.

### Yap siRNA constructs and delivery

Four different human Yap siRNA were designed and synthesized in RNA oligo duplex with dTdT modification on both 5′ and 3′ ends (Life Technology, Grand Island, NY). Sense strand sequences are as follows: siYAP: #1: 5′-GACAUCUUCUGGUCAGAGA-3′; siYAP #2: 5′-GCCACCAAGCUAGAUAAAGAA-3′; siYAP #3: 5′-GCUCAUUCCUCUCCAGCUU-3′; siYAP #4: 5′-CCCAGUUAAAUGUUCACCAAU-3′. RISC-free RNAi control was purchased from GE Healthcare Dharmacon (D-001220–01, Pittsburgh, PA). The siRNAs were reconstituted and stored according to vendor protocols. Lipofectamine RNAiMax was used to transfect RNAi oligos into HUVEC in culture (Life Technology).

### Antibodies

Primary antibodies used in western blots were as follows: rabbit CASPASE-3 (#9662, Cell Signaling), rabbit cleaved CASPASE 3 (#9661, Cell Signaling), rabbit CDC6 (#3387, Cell Signaling), mouse GAPDH (G8140, US Biological, Swampscott, MA), rabbit MCM4 (BD Pharmingen, San Jose, CA), mouse PCNA (sc-56, Santa Cruiz Biotechnology), rabbit and mouse Yap (sc-15407 and sc-101199, Santa Cruz Biotechnology, Santa Cruz; #4912 Cell Signaling, Danvers, MA; #2060, Epitomics, Burlingame, CA). All antibodies were used at 1:1000 except GAPDH which was 1:2000.

Secondary antibodies were purchased from Jackson ImmunoResearch (West Grove, PA). HRP-conjugated Donkey anti-rabbit IgG (711–036–152) and HRP-conjugated Donkey anti-mouse IgG (715–035–150) were used at 1:4000 to 1:5000 for western blots.

### BrdU immunofluorescence assay

BrdU stock was commercially purchased and added to cells at the concentration specified in vendor’s protocol for 1–2 hours (BD Bioscience, San Jose, CA). Cells were fixed with 4% paraformaldehyde (Electron Microscopy Sciences, Hatfield, PA) and washed with PBS. HCl was used to denature DNA for 20 minutes at room temperature (3N HCl, 0.5% Tween-20 and ddH2O) and neutralized with 0.1M sodium borate, pH 8.5. 1:50 Monoclonal BrdU antibody (MoBu-1, Life Technology, Grand Island, NY) and 488 conjugated donkey anti-mouse secondary (Jackson Immunoresearch, West Grove, PA) were incubated with cells at 37°C for 30min, respectively. The fluorescence images were taken on a standard fluorescence microscope and analyzed on ImageJ (Fiji).

### BrdU flow cytometry

BrdU stock was commercially purchased and added to cells at the concentration specified in vendor’s protocol for 1–2 hours (BD Bioscience, San Jose, CA). The incubated cells were processed either with BD Pharmigen FITC BrdU Flow Kit (559619) or HCl-based method. For the latter approach, cells were trypsinized and fixed with either 70% ethanol overnight in -20°C. Fixed cells were washed in washing buffer (1% BSA, 0.25% Triton-X and 1X PBS). DNA was denatured with HCl solution (3N HCl, 0.5% Tween-20 and ddH2O) for 20 minutes at room temperature. Denatured cells were then neutralized with 0.1M sodium borate, pH 8.5 for 2 minutes at room temperature. Alexa 488 conjugated BrdU antibody (B35139, Life Technology, Grand Island, NY) was added to cells at 1:50 dilution in wash buffer for 1 hour at room temperature in the dark. Cells were treated with propidium iodide for DNA counter staining and analyzed on FACS machine directly.

BrdU profiles were collected on BD FACS Calibur using Cell Quest software (BD Bioscience, San Jose, CA). BrdU was collected in FL1 channel (530/30nm) and propidium iodide in FL2 channel (585/42nm). The results were analyzed on FlowJo (Treestar, Ashland, OR) and Excel (Microsoft, Richmond, VA).

### Real-time PCR

RNA was extracted using RNease or miRNeasy kit (Qiagen, Valencia, CA) and reverse transcribed into cDNA with iScript Advanced cDNA Synthesis kit (Bio-Rad, Hercules, CA). The qPCR is performed using SyBr Green (Applied Biosystems, Bedford, MA) with ABI 7900HT or SsoAdvanced SyBr Green with CFX384 (Bio-Rad, Hercules, CA). See S3 Table for primer sequences.

### Microarray analysis

RISC-free control, siYAP#1 and siYAP#2 treated HUVECs were collected at 30 hours post siRNA transfection. RNA was extracted using the miRNeasy kit (Qiagen, Valencia, CA) submitted in biological quadruplicates for the microarray analysis. The microarray was performed by University of Pennsylvania Genomic Analysis Core using Affymetrix Human Exon Gene Chip 1.0 (Affymetrix, Santa Clara, CA). The raw array data was processed on Partek software at Penn Bioinformatics Core (Partek, St. Louis, MO). Additional bioinformatics analysis was performed using NIH DAVID Bioinformatics Resources database (http://david.abcc.ncifcrf.gov/) and Broad Institute Gene Set Enrichment Analysis (http://www.broadinstitute.org/gsea/index.jsp). The microarray data have been uploaded to the GEO repository (http://www.ncbi.nlm.nih.gov/geo/query/acc.cgi?acc=GSE61989).

## Supporting Information

S1 Fig Selecting HUVEC and siRNA for YAP function studies (see also Fig. 1). A. Immunoblot (IB) analysis assessing the levels of endogenous YAP and its response to VEGF in protein lysates extracted from different endothelial cell lines. YAP was expressed in HUVEC, HMVEC and HLVEC but not in MLEC. The presence of VEGF did not affect YAP expression in any of the endothelial lines examined. A lower molecular weight YAP band was detected in HLVEC which may be from IgG background or a cell line-specific YAP isoform. HUVEC: human umbilical vein endothelial cells. HMVEC: human microvascular endothelial cells. HLVEC: human liver vein endothelial cells. MLEC: mouse lung endothelial cells. GAPDH: loading control. B. IB analysis control for CASP3 antibody used in Fig. 1C. The siYAP#3 was one of the five siYAPs generated that consistently elicited extensive apoptotic responses in HUVECs. The CASP3 antibody was capable of detecting both full length and cleaved CASP3 in protein lysates extracted from HUVECs 48 hours post siRNA transfection. C. IB analysis for siRNA knockdown efficiency assessment in HUVEC and HeLa. Protein lysates were collected at 1, 2 or 3 days after siRNA transfection for IB analysis. The siYAPs was highly efficient in both cell lines starting 24 hours post knockdown. No endogenous YAP was detected at 48 and 72 hours after siRNA transfection. GAPDH: loading control.
(PDF)

S2 Fig *YAP* knockdown leads to increase in cell sizes and loss of cycling cells (see also Fig. 1). A. Forward scatter (FSC) plot of controls and YAP-KD HUVECs from Day 0 to Day 5 post siRNA transfection in Fig. 1A. The FSC measurements of YAP-KD HUVECs were significantly higher than that of the control groups, suggesting that YAP-KD cells were likely larger in their sizes. *: p-value < 0.002. B. Immunofluorescence (IF) of BrdU stained cells used for quantification in Fig. 1A. Cells were first incubated with BrdU for 30 min and then fixed and stained for BrdU and DAPI. Representative images were used for visualization. The siYAP treated HUVECs contained significantly fewer BrdU+ cells than the control groups. DAPI: DNA marker.
(PDF)

S3 Fig *YAP* deletion leads to G1 accumulation in HUVECs (see also Fig. 2). HUVEC BrdU FACS analysis of cell cycles in controls and YAP-KD cells. The % of cells in G1, S or G2/M was calculated as the ratio of the number of cells in each phase over the total number of cells counted. Y-axis: BrdU intensity (in units of 10), X-axis: propidium iodide (PI) marking DNA content; 2N = 400 arbitrary units, 4N = 800 arbitrary units. FACS gatings were annotated on the graphs. The images were representative results (N = 3). YAP-KD HUVECs begun to accumulate in G1 and to lose S-phase cells starting on Day 2 after siRNA transfection.
(PDF)

S4 Fig YAP is not essential to HeLa proliferation (see also Fig. 2). A. HeLa BrdU FACS analysis of cell cycles in controls and YAP-KD cells. Y-axis: BrdU intensity (in units of 10), X-axis: propidium iodide (PI) marking DNA content; 2N = 400 arbitrary units, 4N = 800 arbitrary units. FACS gatings were annotated. The images were representative results (N = 3). B. Quantification of Part (A). The % of cells in G1, S or G2/M was calculated as the ratio of the number of cells in each phase over the total number of cells counted. The controls and YAP-KD HeLa cells exhibited normal cell cycle profiles from Day 1 to Day 3 post siRNA treatment, suggesting that YAP is not essential in HeLa proliferation.
(PDF)

S5 Fig HUVEC S-phase progression is YAP-independent (see also Fig. 3). HUVEC BrdU FACS analysis of cell cycles in controls and YAP-KD cells with 6 hours of APH arrest. The acute APH arrest stalled HUVECs in S-phase (6hr APH). HUVECs begun recovery 3 hours after APH washout (Release 3hr). Both control and YAP-KD cells re-initiated early S phase after 3 hours of recovery and mid S phase after 6 hours, which suggests that HUVEC S-phase progression is likely YAP-independent. Y-axis: BrdU intensity (in units of 10), X-axis: propidium iodide (PI) marking DNA content; 2N = 400 arbitrary units, 4N = 800 arbitrary units. FACS gatings were annotated. The images were representative results (N = 3). Red arrow: early S-phase cells. Blue arrow: mid S-phase cells.
(PDF)

S6 Fig YAP is required for HUVEC S-phase initiation (see also Fig. 4). HUVEC BrdU FACS analysis of cell cycles in controls and YAP-KD cells with 24 hours of APH arrest. The 24 hour APH incubation arrested cells predominantly in either G1 or G2/M (APH 24hr). Control HUVECs entered early S phase after 6 hours of APH removal (Release 6hr) and back to normal cell cycling after an additional 18 hours (Release 24hr). In contrast, YAP-KD mutants were unable to initiate S phase at 6 hours post APH washout and exhibited G1 accumulation at 24 hours post recovery. Y-axis: BrdU intensity (in units of 10), X-axis: propidium iodide (PI) marking DNA content; 2N = 400 arbitrary units, 4N = 800 arbitrary units. FACS gatings were annotated. The images were representative results (N = 3). Red arrow: early/mid S-phase cells.
(PDF)

S7 Fig Microarray analysis of YAP deficient HUVECs (see also Fig. 5). A. RT-PCR assessing the level of *YAP* knockdown in HUVECs used for microarray at 30 hours post siRNA transfection. RNA was extracted from HUVECs and reverse-transcribed into cDNA before RT-PCR analysis. In comparison to both control groups, YAP-KD cells have lost approximately 90% of the endogenous *YAP* mRNA at the time of collection. Each condition contained biological quadruplicates (N = 4). B. Venn diagram (left panel) and association plot (right panel) of candidates with expression change above 1.5 fold in both siYAP#1 and siYAP#2. C. Venn diagram explaining the step-wise microarray result analysis. Fold change combination #1 (left): gene expression fold change > 1.5 in siYAP#1 and >1.2 in siYAP#2. Fold change combination #2 (right): gene expression fold change > 1.2 in siYAP#1 and >1.5 in siYAP#2. A significant overlapping (80–95%) in microarray candidate genes was observed between siYAP#1 and siYAP#2 in this step-wise analysis. D. Because the two siYAPs likely elicited different magnitudes of responses in HUVECs, only 70 common targets were generated under the stringent fold change threshold (>1.5 fold, panel B) in the Venn diagram analysis. However, the step-wise analysis from (panel C demonstrated that there is still significant overlapping between siYAP#1 and siYAP#2 array candidates. Linear associate plot of these 70 genes found that they were predominantly down-regulated with a small subset up-regulated. Only 4% were differentially regulated representing likely the off target effects of siRNAs.
(PDF)

S8 Fig Loss of *YAP* leads to decreased MCM4 and CDC6 protein levels (see also Fig. 5). HUVEC IB analysis validating the microarray results using antibodies against MCM4 and CDC6. A significant loss of both MCM4 and CDC6 proteins was detected in siYAP treated HUVECs, which is consistent with the microarray analysis and cell cycle profiling results. GAPDH: loading control.
(PDF)

S1 Table *YAP* microarray analysis candidate gene lists with gene expression change of 1.2 fold or above with a p-value less than 0.05 and a false discovery rate below 0.1. The “Down 1.2” tab contains all common genes between both siYAP#1 and siYAP#2 HUVECs with decreased expression change of 1.2 fold or more. The “Up 1.2” tab contains all common genes between both siYAP#1 and siYAP#2 HUVECs with increased expression change of 1.2 fold or more.
(XLS)

S2 Table *YAP* microarray analysis candidate gene lists with gene expression change of 1.5 fold or above with a p-value less than 0.05 and a false discovery rate below 0.1. The “Down 1.5” tab contains all common genes between both siYAP#1 and siYAP#2 HUVECs with decreased expression change of 1.5 fold or more. The “Up 1.5” tab contains all common genes between both siYAP#1 and siYAP#2 HUVECs with increased expression change of 1.5 fold or more.
(XLS)

S3 Table RT-PCR primer sequences for microarray result validation. (XLS)
