# Supplementary figures and images for: YAP Regulates S-Phase Entry in Endothelial Cells
Source: PLoS One. 2015 Jan 30;10(1):e0117522. doi: 10.1371/journal.pone.0117522 (PMC4312014; doi:10.1371/journal.pone.0117522)

Supp. Fig. 1 (see also Fig. 1)

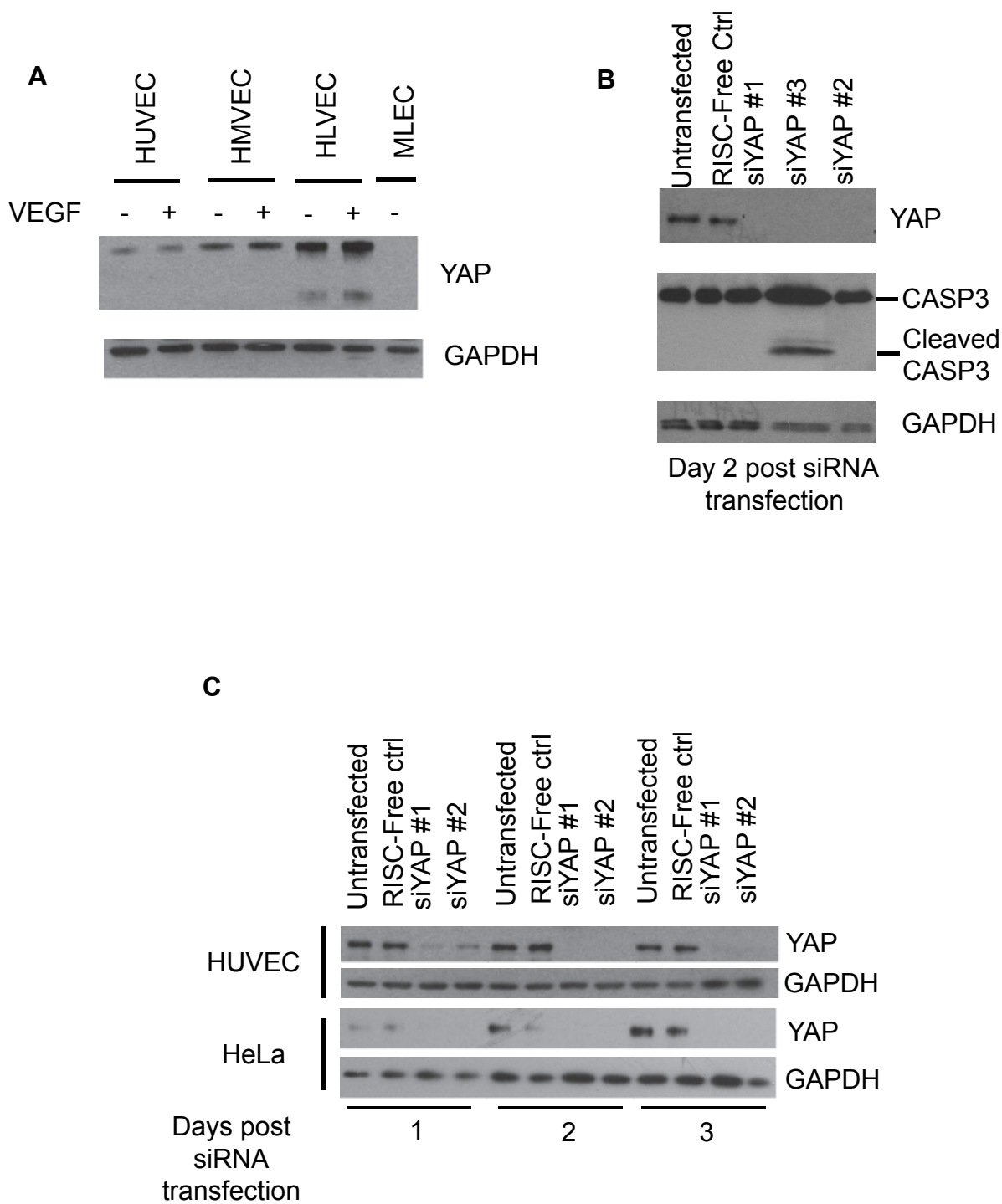

Supplement: S1 Fig — A. Immunoblot (IB) analysis assessing the levels of endogenous YAP and its response to VEGF in protein lysates extracted from different endothelial cell lines. YAP was expressed in HUVEC, HMVEC and HLVEC but not in MLEC. The presence of VEGF did not affect YAP expression in any of the endothelial lines examined. A lower molecular weight YAP band was detected in HLVEC which may be from IgG background or a cell line-specific YAP isoform. HUVEC: human umbilical vein endothelial cells. HMVEC: human microvascular endothelial cells. HLVEC: human liver vein endothelial cells. MLEC: mouse lung endothelial cells. GAPDH: loading control. B. IB analysis control for CASP3 antibody used in Fig. 1C. The siYAP#3 was one of the five siYAPs generated that consistently elicited extensive apoptotic responses in HUVECs. The CASP3 antibody was capable of detecting both full length and cleaved CASP3 in protein lysates extracted from HUVECs 48 hours post siRNA transfection. C. IB analysis for siRNA knockdown efficiency assessment in HUVEC and HeLa. Protein lysates were collected at 1, 2 or 3 days after siRNA transfection for IB analysis. The siYAPs was highly efficient in both cell lines starting 24 hours post knockdown. No endogenous YAP was detected at 48 and 72 hours after siRNA transfection. GAPDH: loading control. (PDF) [file pone.0117522.s001.pdf]

**Supp. Fig. 2 (see also Fig. 1)**

**A**

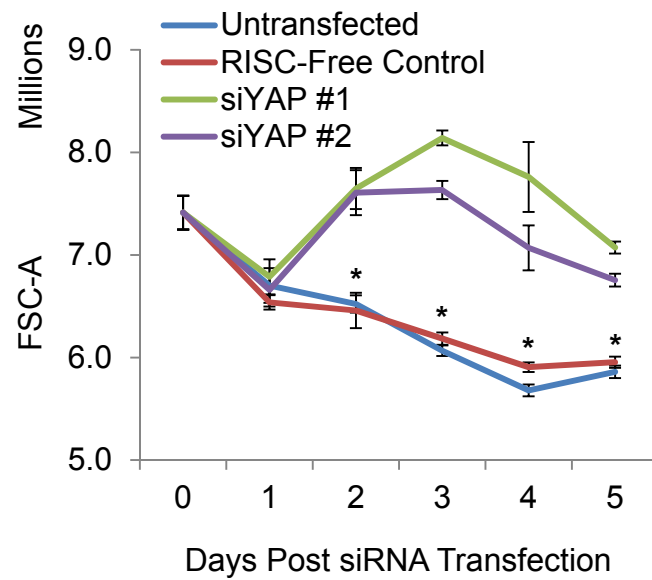

**B**

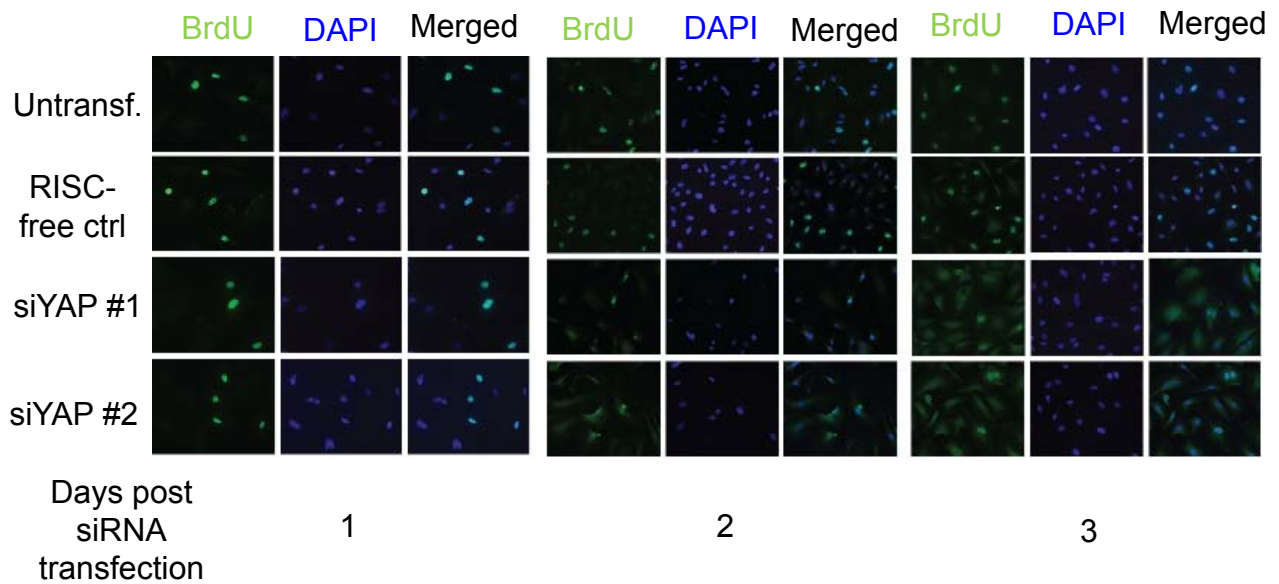

Supplement: S2 Fig — A. Forward scatter (FSC) plot of controls and YAP-KD HUVECs from Day 0 to Day 5 post siRNA transfection in Fig. 1A. The FSC measurements of YAP-KD HUVECs were significantly higher than that of the control groups, suggesting that YAP-KD cells were likely larger in their sizes. *: p-value < 0.002. B. Immunofluorescence (IF) of BrdU stained cells used for quantification in Fig. 1A. Cells were first incubated with BrdU for 30 min and then fixed and stained for BrdU and DAPI. Representative images were used for visualization. The siYAP treated HUVECs contained significantly fewer BrdU+ cells than the control groups. DAPI: DNA marker. (PDF) [file pone.0117522.s002.pdf]

Supp. Fig. 3 (see also Fig. 2)

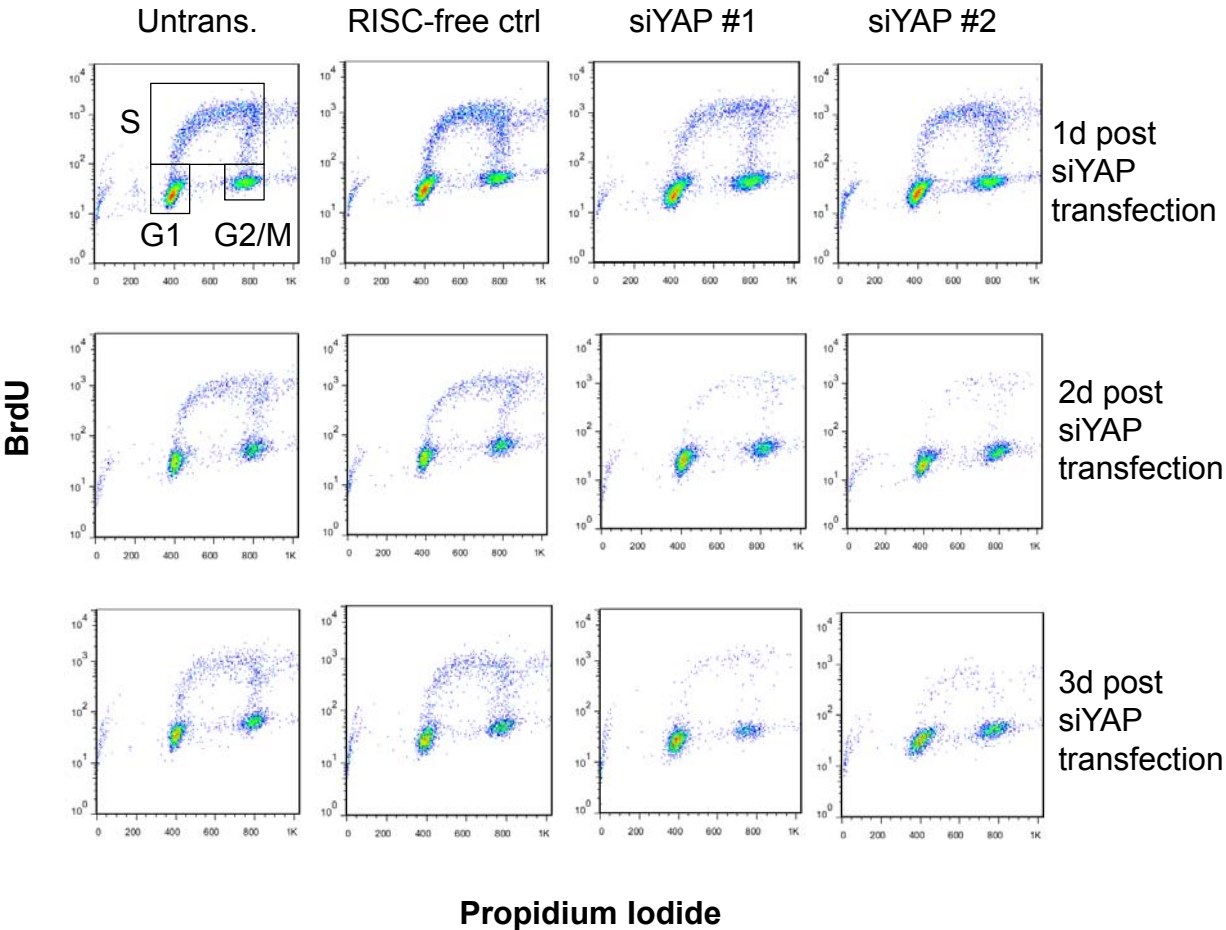

Supplement: S3 Fig — HUVEC BrdU FACS analysis of cell cycles in controls and YAP-KD cells. The % of cells in G1, S or G2/M was calculated as the ratio of the number of cells in each phase over the total number of cells counted. Y-axis: BrdU intensity (in units of 10), X-axis: propidium iodide (PI) marking DNA content; 2N = 400 arbitrary units, 4N = 800 arbitrary units. FACS gatings were annotated on the graphs. The images were representative results (N = 3). YAP-KD HUVECs begun to accumulate in G1 and to lose S-phase cells starting on Day 2 after siRNA transfection. (PDF) [file pone.0117522.s003.pdf]

Supp. Fig. 4 (see also Fig. 2)

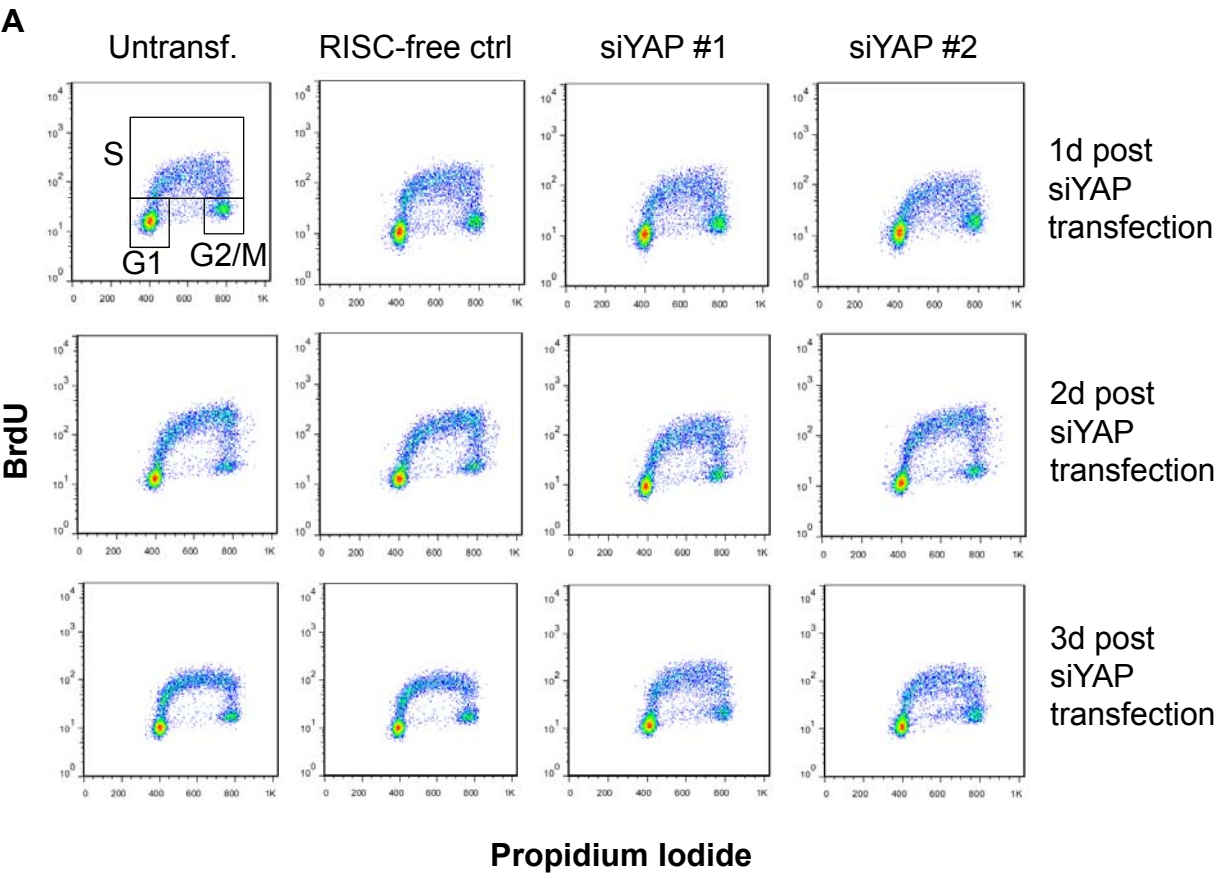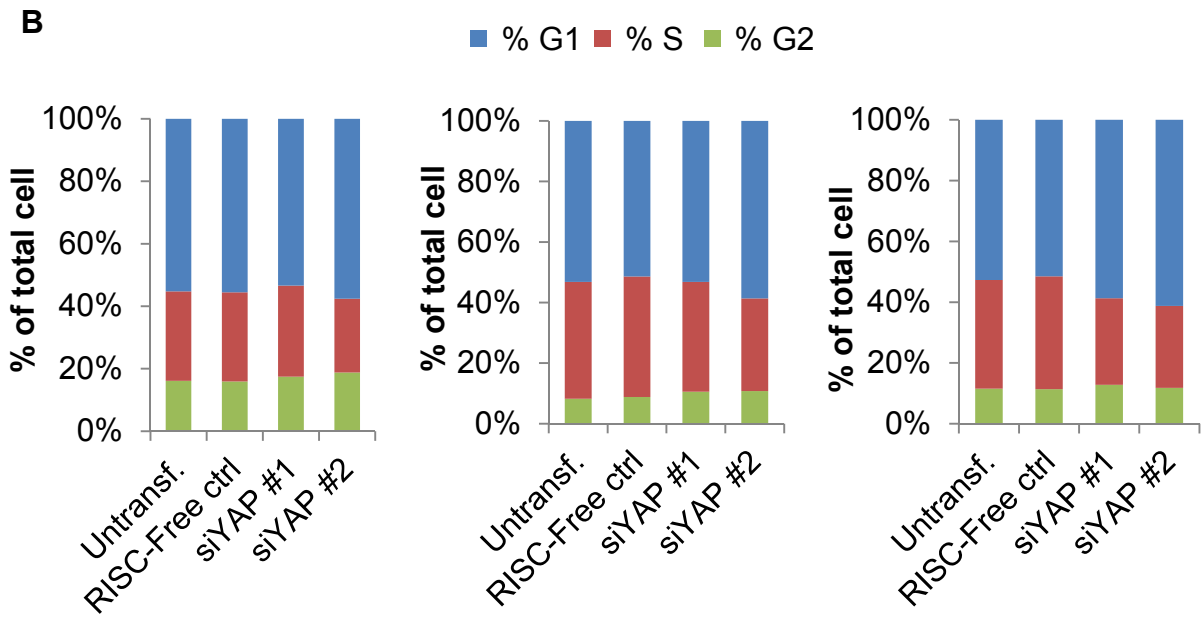

Supplement: S4 Fig — A. HeLa BrdU FACS analysis of cell cycles in controls and YAP-KD cells. Y-axis: BrdU intensity (in units of 10), X-axis: propidium iodide (PI) marking DNA content; 2N = 400 arbitrary units, 4N = 800 arbitrary units. FACS gatings were annotated. The images were representative results (N = 3). B. Quantification of Part (A). The % of cells in G1, S or G2/M was calculated as the ratio of the number of cells in each phase over the total number of cells counted. The controls and YAP-KD HeLa cells exhibited normal cell cycle profiles from Day 1 to Day 3 post siRNA treatment, suggesting that YAP is not essential in HeLa proliferation. (PDF) [file pone.0117522.s004.pdf]

Supp. Fig. 5 (see also Fig. 3)

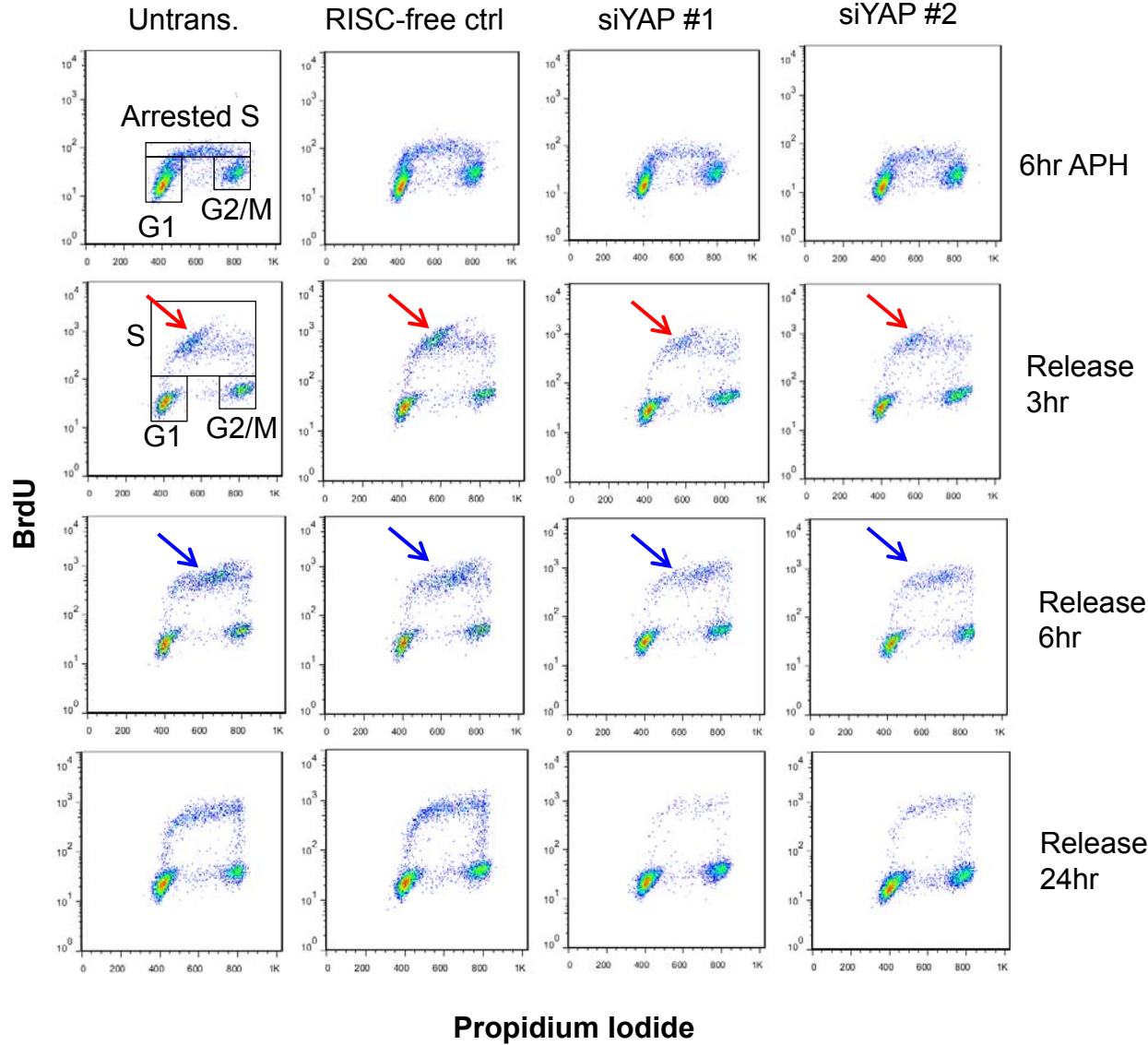

Supplement: S5 Fig — HUVEC BrdU FACS analysis of cell cycles in controls and YAP-KD cells with 6 hours of APH arrest. The acute APH arrest stalled HUVECs in S-phase (6hr APH). HUVECs begun recovery 3 hours after APH washout (Release 3hr). Both control and YAP-KD cells re-initiated early S phase after 3 hours of recovery and mid S phase after 6 hours, which suggests that HUVEC S-phase progression is likely YAP-independent. Y-axis: BrdU intensity (in units of 10), X-axis: propidium iodide (PI) marking DNA content; 2N = 400 arbitrary units, 4N = 800 arbitrary units. FACS gatings were annotated. The images were representative results (N = 3). Red arrow: early S-phase cells. Blue arrow: mid S-phase cells. (PDF) [file pone.0117522.s005.pdf]

**Supp. Fig. 6 (see also Fig. 4)**

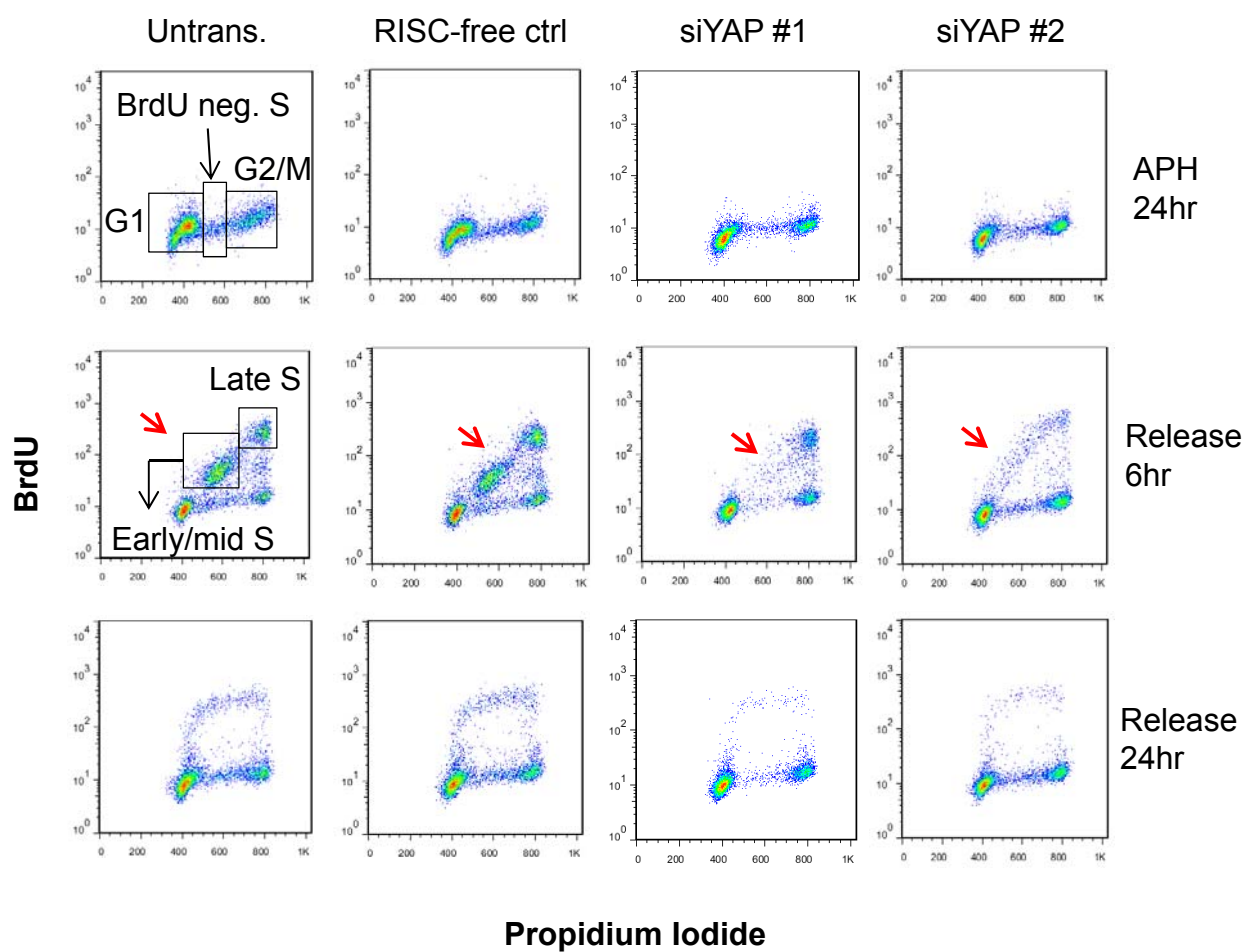

Supplement: S6 Fig — HUVEC BrdU FACS analysis of cell cycles in controls and YAP-KD cells with 24 hours of APH arrest. The 24 hour APH incubation arrested cells predominantly in either G1 or G2/M (APH 24hr). Control HUVECs entered early S phase after 6 hours of APH removal (Release 6hr) and back to normal cell cycling after an additional 18 hours (Release 24hr). In contrast, YAP-KD mutants were unable to initiate S phase at 6 hours post APH washout and exhibited G1 accumulation at 24 hours post recovery. Y-axis: BrdU intensity (in units of 10), X-axis: propidium iodide (PI) marking DNA content; 2N = 400 arbitrary units, 4N = 800 arbitrary units. FACS gatings were annotated. The images were representative results (N = 3). Red arrow: early/mid S-phase cells. (PDF) [file pone.0117522.s006.pdf]

Supp. Fig. 7 (see also Fig. 5)

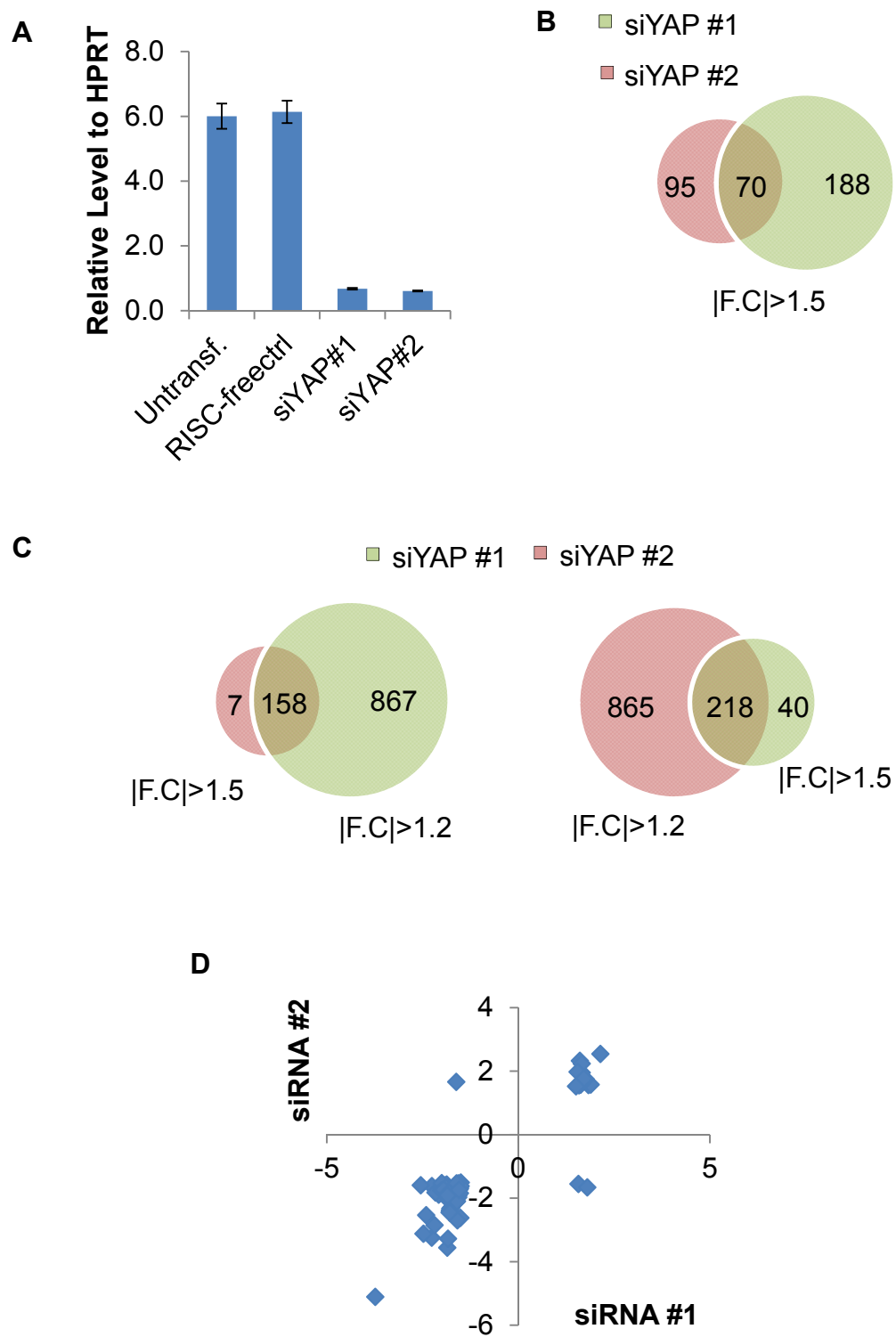

Supplement: S7 Fig — A. RT-PCR assessing the level of YAP knockdown in HUVECs used for microarray at 30 hours post siRNA transfection. RNA was extracted from HUVECs and reverse-transcribed into cDNA before RT-PCR analysis. In comparison to both control groups, YAP-KD cells have lost approximately 90% of the endogenous YAP mRNA at the time of collection. Each condition contained biological quadruplicates (N = 4). B. Venn diagram (left panel) and association plot (right panel) of candidates with expression change above 1.5 fold in both siYAP#1 and siYAP#2. C. Venn diagram explaining the step-wise microarray result analysis. Fold change combination #1 (left): gene expression fold change > 1.5 in siYAP#1 and >1.2 in siYAP#2. Fold change combination #2 (right): gene expression fold change > 1.2 in siYAP#1 and >1.5 in siYAP#2. A significant overlapping (80–95%) in microarray candidate genes was observed between siYAP#1 and siYAP#2 in this step-wise analysis. D. Because the two siYAPs likely elicited different magnitudes of responses in HUVECs, only 70 common targets were generated under the stringent fold change threshold (>1.5 fold, panel B) in the Venn diagram analysis. However, the step-wise analysis from (panel C demonstrated that there is still significant overlapping between siYAP#1 and siYAP#2 array candidates. Linear associate plot of these 70 genes found that they were predominantly down-regulated with a small subset up-regulated. Only 4% were differentially regulated representing likely the off target effects of siRNAs. (PDF) [file pone.0117522.s007.pdf]

**Supp. Fig. 8 (see also Fig. 5)**

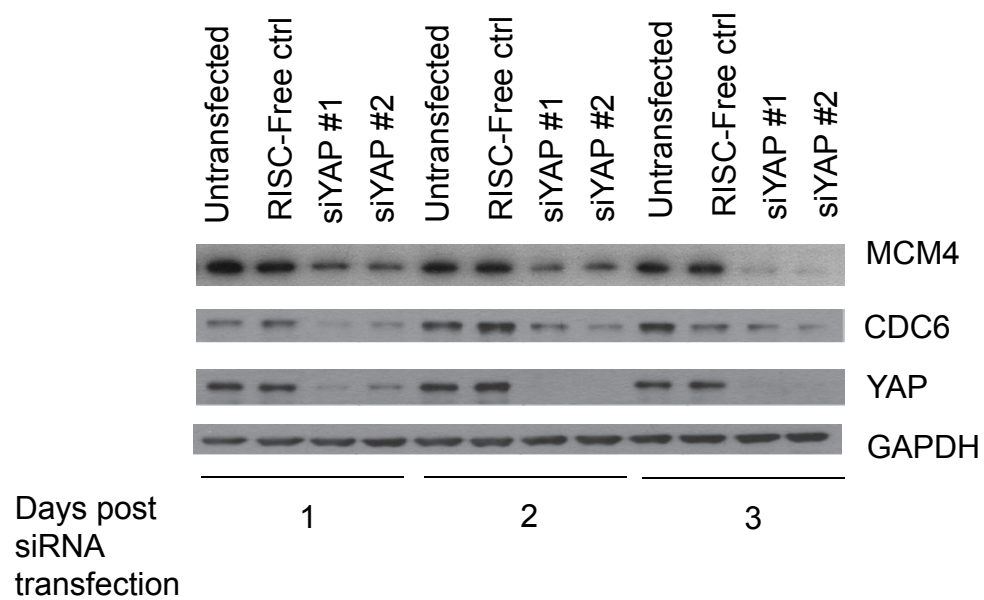

Supplement: S8 Fig — HUVEC IB analysis validating the microarray results using antibodies against MCM4 and CDC6. A significant loss of both MCM4 and CDC6 proteins was detected in siYAP treated HUVECs, which is consistent with the microarray analysis and cell cycle profiling results. GAPDH: loading control. (PDF) [file pone.0117522.s008.pdf]
